# Restoring Functionality: A Case Report on Physiotherapeutic Rehabilitation for L5-S1 Anterolisthesis Management

**DOI:** 10.7759/cureus.56513

**Published:** 2024-03-19

**Authors:** Mansee S Dangare, Nikita Gangwani, Priya Tikhile, Anushka P Bhagwat, Mitushi Deshmukh, Pratik Phansopkar

**Affiliations:** 1 Musculoskeletal Physiotherapy, Ravi Nair Physiotherapy College, Datta Meghe Institute of Higher Education and Research, Wardha, IND

**Keywords:** case report, physiotherapy, quality of life, physiotherapy rehabilitation, lower back pain, bilateral piriformis syndrome, l5-s1 anterolisthesis

## Abstract

Anterolisthesis is a condition where a vertebra in the spine slips forward relative to the vertebra below it. Anterolisthesis is often described in terms of the direction of the slippage and the affected vertebrae, such as L5-S1 anterolisthesis, which indicates the slippage occurring between the fifth lumbar vertebra (L5) and the sacral bone (S1). Anterolisthesis can result from various factors, trauma, or congenital abnormalities. The symptoms associated with anterolisthesis can include lower back pain, stiffness, muscle tightness, and neurological symptoms if the slippage compresses nearby nerves. The piriformis muscle, situated deep within the buttocks, plays a crucial role in this scenario, as its contraction or inflammation can exacerbate the compression of the sciatic nerve, intensifying the pain and discomfort experienced by the individual. Patients with L5-S1 anterolisthesis and bilateral piriformis syndrome commonly report challenges in daily activities involving hip movement, such as walking, sitting, or standing for prolonged periods of time. The combined effects of vertebral slippage and piriformis involvement contribute to altered gait patterns and may lead to difficulties in maintaining a stable and pain-free posture. Effective management often necessitates a comprehensive approach, encompassing physical therapy, pain management strategies, and, in severe cases, surgical intervention. We report a case of a 75-year-old male who complained of pain in his back radiating to both lower limbs with a history of slipping and falling in the bathroom one month prior, sustaining an injury to his back, and who visited the orthopedics department of* *Acharya Vinoba Bhave Rural Hospital (AVBRH), Sawangi, Wardha, where an investigation was done and an X-ray revealed L5-S1 anterolisthesis. Physiotherapy plays a crucial role in reducing pain, improving the range of motion and muscle strength, decreasing muscle tightness, and enhancing the quality of life. The goal of physiotherapeutic rehabilitation for L5-S1 anterolisthesis management is to optimize functional recovery, reduce pain, improve the range of motion and muscle strength, and improve the overall quality of life for individuals with this condition.

## Introduction

Lumbar spondylolisthesis (LS) is a frequent back condition. Anterolisthesis is the displacement of the vertebra compared to the neighboring vertebral column below it, which might be moving ahead, backward, or laterally [1,2]. Spondylolisthesis often denotes a forward translation of an upper vertebra in the sagittal direction with respect to an adjacent lower vertebra. One of the main causes of spinal canal narrowing is lumbar degenerative spondylolisthesis, which is frequently linked to lower spine and leg discomfort [3]. Anterolisthesis is caused by facet articulation degeneration. However, retrolisthesis is caused by intervertebral disc degeneration [4]. With regard to S1, lumbosacral kyphosis is a more relevant morphologic diagnostic indicator than the percentage of L5 anterolisthesis. The correct alignment of the lumbosacral-pelvic architecture is critical in determining the forces that are exerted on both the front and back parts of the lumbar spinal column [5].

Patients with piriformis syndrome may have lower back discomfort that spreads down the buttocks and legs owing to sciatic nerve compression. The patient also complains of soreness when bending forward. Also, because anterior displacement can modify the position of the spine, there may be apparent alterations in gait and posture. Individuals suffering from this ailment may also have sensations of numbness, tingling, or weakness in their legs, which is aggravated by extended sitting or walking [6].

Piriformis syndrome develops when your piriformis muscle compresses the sciatic nerve, causing irritation [7]. Combinations of nerve roots from L4 to S3 make up the sciatic nerve. Several additional diseases that can cause discomfort in the back, buttock pain, and sciatica are included in a possible diagnosis of piriformis syndrome. These diseases include radiculopathy, lumbar spinal stenosis, affliction mediated by the sacroiliac (SI) joint, pain mediated by the hip joint, pain mediated by the facet joint, greater trochanteric pain syndrome, and discomfort intrinsic to the thigh muscles along with tendons and fasciae [8]. When the hip bends, the piriformis muscle acts as a hip abductor, as well as an external rotation of the hip. The piriformis muscle is connected to a nerve composed of branches from the posterior divisions of the ventral rami of S1 [9]. Rehabilitation can assist in reducing posttreatment pain by applying ice and heat to the tense and inflamed piriformis muscle [10]. Regular stretching exercises combined with deep tissue mobilization reduce sciatic nerve compression. Good body position and movement awareness also help to minimize unintentional muscle spasms [11].

## Case presentation

Patient information

A 75-year-old male, teacher by occupation, right-sided dominant, and resident of Yavatmal, complained of pain in his back radiating to both lower limbs. He gave an alleged history of slipping and falling in the bathroom one month prior, sustaining an injury to his back. The pain was sudden onset, sharp shooting, and progressive. He went to a private hospital in Nagpur, where medications were prescribed. He got relief from the medications. But a few days ago, the patient complained of pain, which was worsening day by day while bending and doing daily activities. He visited the orthopedics department of Acharya Vinoba Bhave Rural Hospital (AVBRH), Sawangi, Wardha, where an investigation was suggested, an X-ray was done, and he was diagnosed with L5-S1 anterolisthesis grade 1 with bilateral piriformis syndrome. Then, the patient was recommended for physiotherapy for rehabilitation, which aims to reduce pain, increase the range of motion, reduce muscle tightness, promote full mobility, and improve the quality of life. The complete incident's timeframe is depicted in Table 1.

**Table 1:** Patient timeline AVBRH: Acharya Vinoba Bhave Rural Hospital

Incidents	Occurrence
Slipping and falling in the bathroom	17/10/2023
Visited a private hospital in Nagpur	20/10/2023
Visited at AVBRH	17/11/2023
The start date of physiotherapy	17/11/2023

Clinical findings

On musculoskeletal examination, the patient was conscious and oriented. Before beginning the examination, the patient's verbal consent was obtained. He was examined in a standing position. A spinal examination revealed no anomalies in the upper extremities. On palpation, the modified Schober test and Patrick's test were positive. The patient's sensory evaluation showed that all dermatomal levels were intact, that he had peripheral neuropathy with tingling sensations, and that he could regulate his bowel and bladder sphincters regularly. The pre- and post-physiotherapy intervention range of motion of lumbar flexion and extension is given in Table 2, side (lateral) lumbar flexion in Table 3, and lumbar manual muscle testing in Table 4.

**Table 2:** Pre- and post-physiotherapy intervention range of motion by modified Schober test

Joint	Pre-physiotherapy intervention	Post-physiotherapy intervention
Lumbar flexion	3 cm	8 cm
Lumbar extension	2 cm	5 cm

**Table 3:** Range of motion of side (lateral) lumbar flexion Rt, right; Lt, left

Joint	Rt active	Rt passive	Lt active	Lt passive
Side (lateral) lumbar flexion	2 cm	3 cm	2 cm	3 cm

**Table 4:** Lumbar manual muscle testing Grade 0, no contractile activity; Grade 1, trace (contractile activity is detectable but no movement); Grade 2, poor (patients complete the partial range of motion); Grade 3, fair (patients complete the range of motion); Grade 4, good (patient can come to the end position but may waver or display some signs of effort); Grade 5, normal (back extensor muscles can quickly come to the end position and hold that position without evidence of significant effort)

Manual muscle testing	Grades
Lumbar flexors	2/5
Lumbar extensors	2/5
Side (lateral) lumbar flexors	2/5
Lumbar spine rotators	2/5

Diagnostic investigations

**Figure 1:**
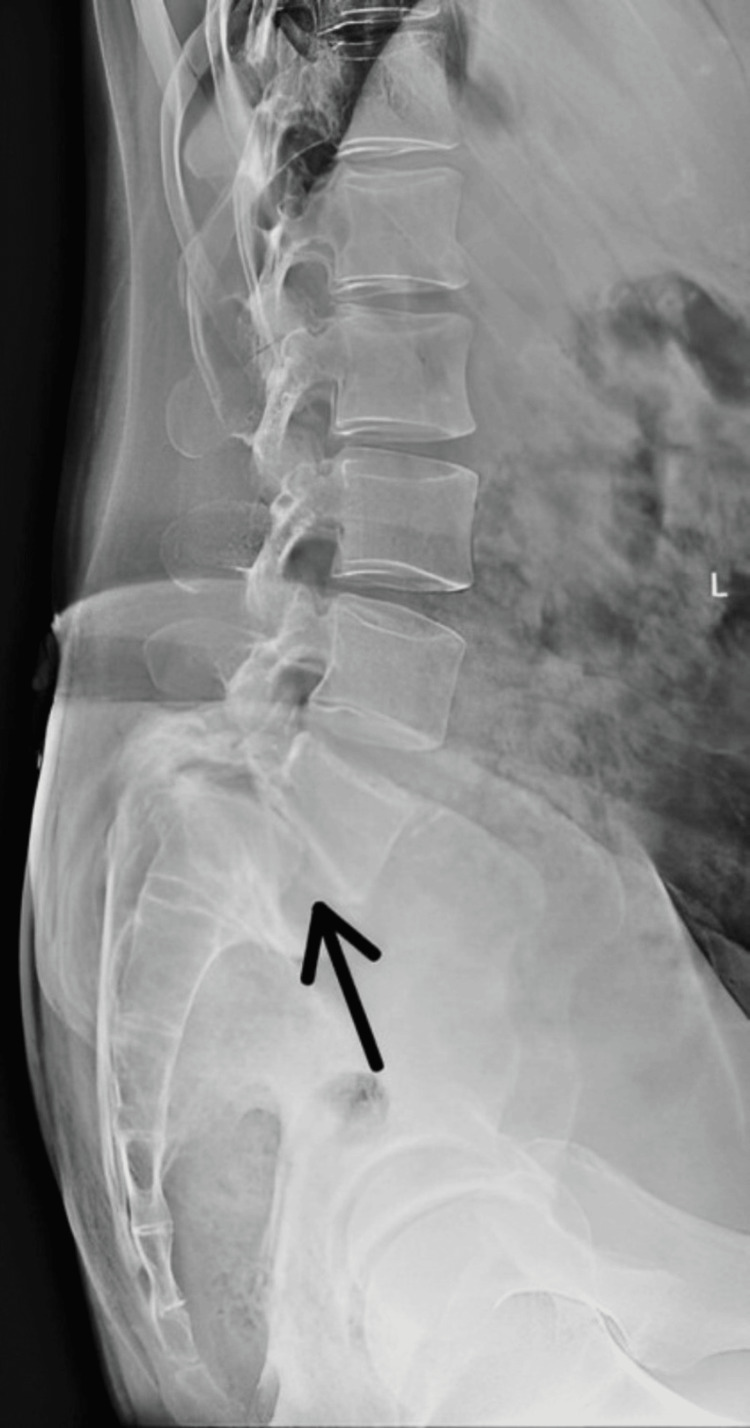
Lumbar spine X-ray The black arrow indicates L5-S1 anterolisthesis

The X-ray was done in lateral view, which shows L5-S1 anterolisthesis as shown in Figure 1.

Therapeutic intervention

**Figure 2:**
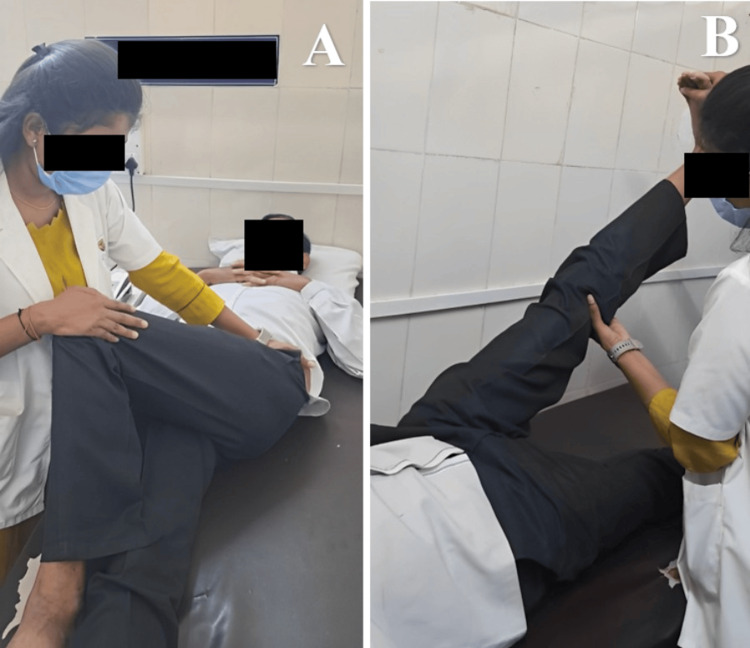
Patient receiving stretching (A) Piriformis stretching of the left side. (B) Hamstring stretching of the left side

The goal-oriented physiotherapy protocol is given in Table 5. Physiotherapy sessions are shown in Figure 2.

**Table 5:** Goal-oriented physiotherapy protocol MET, muscle energy technique; reps, repetitions

Goals	Therapeutic intervention	Treatment protocol
Patient education	A patient is informed about their disease, as well as the importance and advantages of physical rehabilitation	Education was given to the patient to do exercises daily and explaining about do's and don'ts
To reduce pain	Hot pack	Twice a day
	Interferential therapy (four-pole vector)	For 10 minutes
To increase muscle strength	MET	Ten reps in one set with five-second holds
To increase the range of motion	McKenzie exercises	Ten reps in one set
To decrease tingling sensation	Neural mobilization of sciatic nerve bilaterally	Three reps in three sets with 30-second holds
To decrease muscle tightness	Passive piriformis stretching to bilateral lower limbs	Ten reps in one set
	Hamstring stretching to bilateral lower limbs	Ten reps in one set
To reduce tone and trigger point	Integrated neuromuscular inhibition technique using Ruddy's reciprocal antagonist facilitation method	Ten reps in one set

Outcome measures

Table 6 shows pre- and post-physiotherapy intervention outcome measures, and Table 7 shows manual muscle testing post intervention.

**Table 6:** Pre- and post-physiotherapy outcome measures

Serial number	Outcome measures	Pre-physiotherapy intervention	Post-physiotherapy intervention
1	Numerical pain rating scale	8/10	2/10
2	Oswestry Disability Index	70% out of 100	20% out of 100
3	Lower extremity functional scale	45/80	75/100

**Table 7:** Manual muscle testing post intervention Grade 0, no contractile activity; Grade 1, trace (contractile activity is detectable but no movement); Grade 2, poor (patients complete the partial range of motion); Grade 3, fair (patients complete the range of motion); Grade 4, good (patient can come to the end position but may waver or display some signs of effort); Grade 5, normal (back extensor muscles can quickly come to the end position and hold that position without evidence of significant effort)

Serial number	Manual muscle testing	Grades
1	Lumbar flexors	4/5
2	Lumbar extensors	4/5
3	Side (lateral) lumbar flexors	4/5
4	Lumbar spine rotators	4/5

The pre-intervention outcome was taken on day 1, and follow-up will take after six months of conservative physiotherapy management. After regular physiotherapy, patients show notable improvement in pain scores, range of motion, muscular strength, and functional status. Regular rehabilitation sessions were recommended to optimize long-term outcomes and prevent recurrence.

## Discussion

A vertebra translating relative to the vertebra below with no significant damage or alteration to the pars interarticularis is known as spondylolisthesis. The inappropriate distribution of weight combined with soft tissue flexibility and instability over a long period of time causes the posterior annular fibers of the intervertebral disc to buckle and cause severe joint movement [12]. If the patient's ability to do everyday tasks is unaffected, people with grade 1 or 2 spondylolisthesis may generally receive conservative treatment. It is possible to have excellent outcomes. Support, movement limitations, and physical treatment are used as conservative therapeutic techniques for very acute or early spinal degeneration [13]. Spondylolisthesis comes in five different forms: dysplastic, isthmic, degenerative, traumatic, and pathological [14]. There are only around 100 examples of traumatic spondylopelvic separation across the L5-S1 disc region [15]. The translation may be anterior or posterior [16].

L5-S1 anterolisthesis is most commonly caused by degenerative disc degeneration. Over time, the intervertebral discs connecting the lumbar and sacral vertebrae might wear out, causing spinal instability [17]. Both indicate three-column damage requiring surgical stabilization since it separates the pelvis from the spine. For such an injury, open reduction and internal stabilization with pedicle screws are thought to be the best course of action. Since this is a three-column damage, stability might be ensured by using frontal support. It can be required to use additional fixation, such as pelvic or iliosacral fixation, for more severe or complicated L5-S1 injuries [18].

Along with lumbar bending exercises, thoracic mobilization and lumbar stabilization exercises were also found to be beneficial. Chiropractic treatment seeks to alleviate neck discomfort and instabilities, improve neck movement, and avoid neurological damage. The technique included high-velocity, low-amplitude cervical intervention on limited levels; lengthy axial displacement of the cervical sections above the C5; and post-isometric stretching to lengthen the hypertonic muscle. Flexibility and extension were the workout methods. The lumbar spine was flexed during flexion exercises. These workouts include pelvic tilt, chest-to-thigh posture, and abdominal strengthening (isometric or isotonic). Lumbar extension beyond the neutral position was the outcome of extension exercises. These workouts include hip extension while prone supine and upper back extension [19,20].

## Conclusions

The presented case report highlights the significant role of physiotherapeutic rehabilitation in the comprehensive management of L5-S1 anterolisthesis. Through a tailored and progressive intervention plan, our patient demonstrated significant improvements in pain reduction, range of motion, muscular strength, and overall quality of life. The strategic integration of therapeutic exercises, manual techniques, and targeted stretching not only addressed the biomechanical challenges associated with anterolisthesis but also fostered the restoration of spinal stability and muscular balance. The positive outcome in this instance illustrates the value of a variety of approaches for managing L5-S1 anterolisthesis, in which physiotherapeutic therapies are essential. Our findings suggest that a carefully designed rehabilitation program, personalized to the individual needs and limitations of the patient, can significantly contribute to functional recovery and long-term well-being. As we investigate the potential benefits of physiotherapy in the treatment of spinal disorders, this case provides important evidence for the usefulness of focused rehabilitation techniques such as McKenzie exercises, neural mobilization of sciatic nerve, and integrated neuromuscular inhibition technique using Ruddy's reciprocal antagonist facilitation method in improving the standard of life for people with L5-S1 anterolisthesis.
